# Editorial: Ethnopharmacological Strategies for Drug Discovery Against African Neglected Diseases

**DOI:** 10.3389/fphar.2022.851064

**Published:** 2022-04-21

**Authors:** John O. Igoli, Yanna C. F. Teles, Sunday E. Atawodi, Valerie A. Ferro, David G. Watson

**Affiliations:** ^1^ Strathclyde Institute of Pharmacy and Biomedical Science, University of Strathclyde, Glasgow, United Kingdom; ^2^ Department of Chemistry, University of Agriculture, Makurdi, Nigeria; ^3^ Departamento de Química e Física, Campus II—Universidade Federal da Paraíba (UFPB), João Pessoa, Brazil; ^4^ Biochemistry Department, Faculty of Science, Federal University Lokoja, Lokoja, Nigeria

**Keywords:** neglected tropical disease (NTD), ethnopharmacology, onchocerciaisis, propolis, traditional “African” therapy

The impact of neglected tropical diseases (NTDs) on the health and economy of neglected communities in the developing World is increasing rapidly while gaining little international attention due to the present Covid-19 pandemic. Although there was international funding for global efforts to eliminate or eradicate ten NTDs by 2020, this remains far from being achieved. However, a strategy for combating NTDs has been recently outlined in a roadmap produced by the World Health Organisation (WHO) (https://www.who.int/publications/i/item/9789240010352) which may stimulate further research. NTDs are a group of infections that mainly affect people living in remote rural areas, urban slums or conflict zones. There is a need to build on this momentum, but a key issue, which has been highlighted is the need for increased research efforts and for strengthening capacity in endemic countries for both research and implementation. To date NTD programs have not fully benefitted from the synergy of research and funding despite many of the diseases being co-endemic. NTD programs can be integrated into primary health care services and existing vaccination or micronutrient campaigns, or the school based distribution of drugs to achieve greater coverage and reduce operational costs. Nigeria carries one of the highest burden and diversity of NTDs in sub-Saharan Africa. The aim of the call for the Research topic: “*Ethnopharmacological Strategies for Drug Discovery against African Neglected Diseases*” was to provide information on the diseases in focus and type of studies carried out by scientists working on the control and elimination of these diseases. According to the WHO the common and predominant NTDs are lymphatic filariasis, onchocerciasis, schistosomiasis, soil-transmitted helminthes Loa loa filariasis, and insect-transmitted diseases such as Dengue fever and leishmaniases. The responses show that many institutions are working on NTDs found in the African continent and their capacity could be readily enhanced with training and resources to boost their skills and to increase their range of technical activities and research visibility, which will also help to provide essential technical and laboratory support to the national NTD programs.

Medicinal plants have been described as natural biochemical factories and they remain the main sources of bioactive molecules used to sustain health, and indeed an enormous amount of research is dedicated to finding bioactive or lead compounds from nature. Although, emphasis on lead finding has shifted to rational synthesis and phytomedicines may have been largely forgotten by Western science, this is not the case in Africa where ethnopharmacological practices still exist and they are the mainstay of primary health care. Recently, however, there is a resurgence in the use of natural substances as drugs, and this development has witnessed a large output of research on natural products, especially those with immune-modifying abilities. Plant-derived substances have been reported for their immunomodulatory potentials. Modulation of the immune system, therefore, offers unlimited strategies for therapy and management of diseases. It is important that the knowledge possessed by those using ethnobotanical therapies is not ignored since it is based on years of primary observation. The types of mixtures used in these therapies are difficult to standardize thus would find it difficult to gain a general license in modern pharmaceutical practice. However, this does not mean that they have no validity and perhaps the most important thing that researchers can do in supporting such therapies is to determine that they are not cytotoxic to mammalian cells, thus determining a therapeutic window, and secondarily to validate the proposed activity using appropriate assays. Even without the final goal of isolating a highly bioactive lead compound, as in the case of artemisinin, this would lend important support to the employment of such medicines as currently practiced in the field. There is still much work to do in taking an ethnopharmacological approach by mapping the use of such medicines by local communities and this is also a precursor to potential drug discovery. The success of this approach can be found in the discovery of artemisinin and taxol which are highly effective drugs today.

Four papers involving ethnobotanical studies, bioassays coupled with bioactive compound isolation and a study on propolis were published under the call. The papers were 1) “*Sidastrum paniculatum (L.) Fryxell (Malvaceae): A Promising Source of Bioactive Sulfated Flavonoids Against Aedes aegypti L*” (Marques et al.). 2) “*Bisbenzylisoquinoline Alkaloids of Cissampelos sympodialis with in vitro Antiviral Activity Against Zika Virus*” (da Silva et al.). 3) “*Leishmanicidal Activity of Propolis Collected in the Semiarid Region of Brazil*” (Cavalcante et al.). 4) “*Cytotoxicity and Genotoxicity of Medicinal Plants Used in the Treatment of Malaria in the Greater Mpigi Region in Uganda*” (Schultz et al.). The aims, methodologies and results reported could be harnessed towards drug discovery for the NTDs and their control in the localities.

The four published manuscripts presented studies related to mosquito-transmitted NTDs, which are a great threat to billions of people in tropical regions of the globe. Countries with unplanned urbanization, poor infrastructures and erratic water supply are the most affected, however, researchers have raised the concern that with climate changes other regions could be threatened by mosquito-transmitted NTDs as well. Therefore, it is crucial to increase efforts in the development of treatments and tools to combat these parasites and their vectors.

The topic begins with a paper targeting a mosquito vector that leads to many viral diseases. The manuscript presented the isolation of sulfated flavonoids from an endemic Brazilian plant species. The compounds have showed larvicidal and insecticidal potential against *Aedes aegypti* L., the mosquito known as the most relevant vector for viruses such as chikungunya, dengue, and Zika, that occurs in tropical regions of Africa and other parts of the globe. The strategy focused on vector controlling has been previously recommended by the WHO for the control of schistosomiasis and other NTDs, and may be an interesting alternative or complement to drug treatment.

The second paper also from researchers in Brazil identified the potential of alkaloids of the bisbenzylisopquinoline type to target Zika virus. This virus was first isolated in Uganda in the late 1940s, and in 2016 was declared as an emerging pathogen of great concern to global public health. To date, without an effective therapeutic approach, there is still an unmet need for new antiviral drugs.

The third paper focused on work carried out in Brazil on a Brazilian propolis targeted at treating Leishmaniasis, one of the most relevant NTDs targeted by the WHO. The current leishmaniasis treatment strategy is based on chemotherapy but the occurrence of resistance has raised the need for novel treatments. The chemical complexity of propolis usually includes variable amounts of phenolic acids and esters phenolics, flavonoids, terpenoids, and steroids that are associated to the geographical location. Thus, propolis has been a relevant subject of pharmaceutical interest and the presented paper demonstrated that the studied Brazilian propolis can be useful to obtain leishmanicidal compounds.

The fourth paper carried out in conjunction with researchers from across Europe, the United States and Africa (Uganda) describes 16 medicinal plants used in traditional medicine and a library of 56 extracts that were screened for activity using a heme biocrystallization assay as a pre-screening method prior to follow up with antiplasmodium experiments. This method proved useful in identifying seven potential plant species. The study added scientific evidence to substantiate the safety and effectiveness in traditional use of these plant species. Care was taken to include extraction methods to represent preparation procedures used in traditional use. Importantly, genotoxicity was also carried out to identify the safe use of these plants and to report findings back to traditional healers.

The topic promoted valuable contributions to the research on the NTDs and the call has rekindled awareness on the burden of NTDs in Africa. With climate change there is evidence that these diseases might spread to other parts of the World thus making the development of new treatments even more important. The variety of chemical structures available in nature is larger than that available from chemical synthesis so there remains great potential for tapping into nature to find new lead compounds. This process can be made more productive where existing local knowledge can be used to increase the likelihood of bioactive leads which can also be used in the management of Covid-19 as several medicinal plant extracts active against the virus have been reported.

